# Tissue ionome response to rhizosphere pH and aluminum in tea plants (*Camellia sinensis* L.), a species adapted to acidic soils

**DOI:** 10.1002/pei3.10028

**Published:** 2020-08-10

**Authors:** Hiroto Yamashita, Yusuke Fukuda, Shiori Yonezawa, Akio Morita, Takashi Ikka

**Affiliations:** ^1^ Faculty of Agriculture Shizuoka University Ohya Shizuoka Japan; ^2^ United Graduate School of Agricultural Science Gifu University Yanagito, Gifu Japan

**Keywords:** aluminum, beneficial element, ionome, rhizosphere pH, tea plant

## Abstract

The growth of tea plants (*Camellia sinensis* L.) is promoted by the presence of aluminum (Al), a beneficial element under acidic conditions, but the influence of rhizosphere pH on this interaction is not known. To understand the mechanisms underlying the adaptation to acidic rhizosphere conditions, we evaluated ionome profiles and the effect of pH on tea growth in hydroponic culture. The optimum pH for tea growth was around pH 4.2, and growth was inferior under a pH less than 3.8 or higher than 5.0. Under the optimum pH growth and Al accumulation were markedly stimulated by Al treatment. Al content and accumulation in new and mature leaves and new roots (the predominant tissues that accumulate minerals in tea plants) gradually declined with decrease in pH, especially in new roots. Ionome profiles drastically altered Al treatment, but changes were more pronounced in new roots than in new or mature leaves and did not depend on pH. Although the uptake of most cationic minerals in new roots was decreased by Al treatment, cationic mineral contents in new and mature leaves were not decreased by Al. In contrast to other plant species, the content and accumulation of manganese, despite it being a cationic nutrient, were significantly increased by Al treatment. These results indicated that one role of Al as a beneficial element was to maintain the shoot nutrient status by effectively utilizing Al‐limited elements in the roots.

## 1 INTRODUCTION

Acidic soils account for approximately 50% of the world’s potentially arable lands but significantly limit crop production (Kochian, Hoekenga, & Piñeros, 2004). Many crops grow poorly in acid soils because of acid soil syndrome, which consists of multiple stress factors, including phytotoxicity to excess ions such as aluminum (Al^3+^), protons (H^+^), and manganese (Mn^2+^), and/or deficiencies of essential minerals such as calcium (Ca), magnesium (Mg), and phosphorus (P) (Kochian et al., 2004). In particular, Al^3+^ stress causes inhibition of nutrient uptake or transport (Lee & Pritchard, 1984; Mariano & Keltjens, 2005) and suppression of root growth (Kinraide, 2003); therefore, Al^3+^ rhizotoxicity is an important factor limiting plant productivity. In addition, excess Al^3+^ and H^+^ compete with other cations for apoplastic binding sites (Horst, Wang, & Eticha, 2010) and activities at the plasma membrane surface (Kinraide, 1994; Kinraide, Ryan, & Kochian, 1992), and also affect the cellular homeostasis of a variety of ions, such as H^+^ (Babourina & Rengel, 2009; Bose, Babourina, Shabala, & Rengel, 2010a), Ca^2+^ (Plieth, Sattelmacher, Hansen, & Knight, 1999; Rengel & Zhang, 2003), K^+^ (Bose, Babourina, Shabala, & Rengel, 2010b), and Mg^2+^ (Bose, Babourina, & Rengel, 2011; Bose, Babourina, Shabala, & Rengel, 2013).

A number of plant species can accumulate Al^3+^ at high concentrations without showing the symptoms of Al^3+^ toxicity. These Al accumulator species, for example *Melastoma malabathricum* L., buckwheat (*Fagopyrum esculentum* Moench) and tea plants (*Camellia sinensis* L.), apparently alleviate its toxicity by complexing Al with ligands, such as organic acids, or by isolating the Al within Al‐insensitive sites, namely the vacuoles (Ma, Zheng, Matsumoto, & Hiradate, 1997; Matsumoto, Hirasawa, Seiichiro, & Takahashi, 1976; Nagata, Hayatsu, & Kosuge, 1992; Watanabe & Osaki, 2002). Al can have beneficial effects on the growth of species that are well adapted to acid soils (Osaki, Watanabe, & Tadano, 1997).

Tea plants grow well in acidic soils because large amounts of Al can accumulate throughout the plant, especially in mature leaves, but stimulates rather than inhibits growth (Ghanati, Morita, & Yokota, 2005; Konishi, Miyamoto, & Taki, 1985; Matsumoto et al., 1976; Morita, Yanagisawa, Takatsu, Maeda, & Hiradate, 2008; Sun et al., 2020). In particular, Al promotes new root growth through maintenance of DNA integrity in root meristematic cells (Sun et al., 2020). Hence, Al is considered to be a beneficial element for tea plants. The growth of tea plants may be stimulated by Al‐induced increase in the activities of antioxidant enzymes, resulting in enhanced membrane integrity and delayed lignification and aging (Ghanati et al., 2005). Hajiboland, Bahrami‐Rad, Barceló, and Poschenrieder (2013) reported that tea plants showed increased antioxidant defenses and a higher photosynthesis rate mediated by Al. Binding of Al to cell wall‐bound phenolic acids would reduce their availability for subsequent enzymatic reactions and might lead to lower lignin content (Hajiboland, Bastani, Bahrami‐Rad, & Poschenrieder, 2015). However, the detailed roles of Al as a beneficial element for tea plants remain unknown.

To improve the yield and quality of tea leaves, tea fields, especially in Japan, tend to receive higher rates of nitrogen (N) fertilizer than other vegetable crops, generally as ammonium sulfate and sometimes exceeding 1000 kg N ha^−1^ year^−1^ (Akiyama, Yan, & Yagi, 2006; Tokuda & Hayatsu, 2004). Heavy use of ammonium sulfate also causes soil acidification as a result of the accumulation of sulfate ions and nitrification (Tachibana, Yoshikawa, & Ikeda, 1995), sometimes leading to such strongly acidic soil with a pH less than 3.0 (Tokuda & Hayatsu, 2004). It is considered that the optimum soil pH for tea cultivation is around pH 4–5, but many tea fields do not meet this standard. As mentioned above, the soil pH also affects the plant response to Al. To achieve sustainable and stable tea cultivation, it is necessary to establish a balance between changes in the degree of Al activity in response to rhizosphere pH changes and tea growth.

The ionome is defined as the mineral nutrient and trace element composition of an organism, representing the inorganic component of cellular and organismal systems (Salt, Baxter, & Lahner, 2008). Ionomics involves quantitative measurement of the elemental composition of organs or tissues and requires the application of high‐throughput elemental analysis technologies using inductively coupled plasma‐atom/optical emission spectrometry (ICP‐AES/OES), ICP‐mass spectrometry (ICP‐MS), X‐ray fluorescence, and neutron activation analysis, and their integration with bioinformatic analysis (Salt et al., 2008). Ionomics is a useful tool to understand physiological processes because plants first perceive minerals in the rhizosphere, and alteration in any processes that transport inorganic ions from the soil solution to the plant body may affect the plant’s ionome (Baxter et al., 2008). Multivariate ionomic signatures were established to define physiological responses such as iron (Fe) and P homeostasis (Baxter et al., 2008). Furthermore, dynamic alterations in the ionome have been confirmed in response to environmental factors including temperature (Quadir, Watanabe, Chen, Osaki, & Shinano, 2011), salt stress (Wu et al., 2013), and N status (Chu et al., 2016).

As mentioned, Al stress can affect the cellular homeostasis of various ions (Babourina & Rengel, 2009; Bose et al., 2010a, 2010b, 2011, 2013; Plieth et al., 1999; Rengel & Zhang, 2003). For an Al accumulator species, it is possible that ion homeostasis is optimized to maintain or promote growth while accumulating Al. In the present study, we studied the effects of acidic pH and Al on the growth and tissue ionome dynamics of tea plants in hydroponic culture to determine the optimum rhizosphere pH and investigate the beneficial roles of Al. The results showed that the alteration to ionome profiles in tea plants caused by Al were not dependent on pH.

## 2 MATERIALS AND METHODS

### 2.1 Plant materials and hydroponic culture

Hydroponic culture of tea plants was conducted under ambient light in an unheated greenhouse (120 m^2^) at Shizuoka University (Shizuoka, Shizuoka, Japan) under an average temperature of 20°C in the spring season (late March to late June) of 2017 and 2018. A slight modification of the culture method described by Konishi et al. (1985) was used. One‐year‐old rooted tea cuttings of “Yabukita,” a leading Japanese green tea cultivar, were transplanted to Wagner pots (1/2000 a; three individuals per pot) containing 12 L tap water adjusted to pH 4.2, and continuously aerated. After 1 week, standard nutrient solutions containing 400 μM Al, prepared from Al_2_(SO_4_)_3_‧14‐18H_2_O, at pH 4.2 (Konishi et al., 1985) was supplied stepwise for 1 week each at 1/5, 1/2, and full strength to adapt the plants to the hydroponic system. The following experiments were subsequently performed. An overview of the hydroponic experiments performed in this study is shown in Figure S1.

An initial hydroponic experiment (in spring of 2017) was performed to evaluate broadly the effects of pH and the presence of Al on plant growth. Plants were transferred to nutrient solutions adjusted using H_2_SO_4_ to various pH values, namely pH 2.8, 3.2, 3.8, 4.2, 4.8, 5.2, 5.8, 6.5, and 7.5, with or without 400 μM Al, prepared from Al_2_(SO_4_)_3_‧14‐18H_2_O. Each experiment was conducted using three biological replicates. The solutions were replaced at 2‐day intervals to maintain the pH. After 5 weeks, tea plants were harvested following the methods described by Morita et al. (2008). At harvest, the roots were immersed in water at pH 3.0 (adjusted with H_2_SO_4_) for 3 min to remove Al absorbed on the root surface. After washing with deionized water, the plants were divided into leaves, stems, and roots, and each part was further separated into new and mature parts: new parts were those that had emerged during treatment, and mature parts were those that were present at the start of treatment. Thus, the growth of new shoots, comprising new leaves and stems, and new roots were evaluated as the growing parts. Each sample was weighed fresh, then freeze‐dried and re‐weighed to determine the dry weight (DW).

The second hydroponic experiment (in spring of 2018) was performed to evaluate in detail the effects of pH and Al on the growth and ionome profile of new roots and leaves. Plants were precultivated under aforementioned standard hydroponic conditions at pH 4.2 with 400 μM Al until the first new shoots had developed five leaves, and then the plants were grown for approximately 2 months to investigate the effects of different treatments on new roots. The first new shoots with five leaves were excised leaving the lowest two leaves, and the plants were transferred to hydroponic solutions adjusted to various pH values from 3.0 to 5.8 in pH 0.2 increments with or without 400 μM Al. The plants were sampled using the same methods as described for the first growth experiment. The freeze‐dried samples were ground into a fine powder then stored in a desiccator at room temperature until analysis. The subsequent mineral analyses were conducted using the samples grown at pH 3.0–5.2 for new leaves and roots, and pH 3.0–5.8 for mature leaves, as the predominant tissues that accumulate minerals in tea plants. New leaves and roots of plants cultured under pH 5.4–5.8 could not be analyzed because the plants did not grow under these extreme conditions.

### 2.2 Mineral quantification

Fine powder (50 mg) of freeze‐dried samples was digested in 2 ml of 60% HNO_3_ at 110°C in DigiTUBE® tubes (SCP SCIENCE, Québec, Canada) for approximately 2 hr. Once the samples had cooled, 2 ml of 60% HClO was added and the samples were heated at 110°C for a further approximately 2 hr. Once digestion was completed, the samples were cooled and made up to a volume of 10 ml with ultra‐pure water. The total concentration of the following 13 elements was measured, based on selected specific wavelengths using an ICP‐OES (iCAP 7400; Thermo Fisher Scientific, Waltham, MA, USA): Al 396.152 nm, Fe 259.940 nm, sodium (Na) 589.592 nm, boron (B) 249.773 nm, P 213.618 nm, sulfur (S) 180.731 nm, silicon (Si) 251.611 nm, Ca 315.887 nm, copper (Cu) 324.754 nm, K 766.490 nm, Mg 285.213 nm, Mn 257.610 nm, and zinc (Zn) 206.200 nm.

Total carbon (C) and N were measured by dry combustion using a Vario MAX cube (Elementar, Hanau, Germany) with aspartic acid as a standard.

### 2.3 Statistical analyses

Plant mineral status was evaluated as mineral content (mg/g DW) and mineral accumulation (mg/plant). Mineral accumulation (mg/plant) was calculated from the mineral content (mg/g DW) and the plant tissues dry weight (g DW). Significant differences in growth, mineral contents, and mineral accumulation between pH and Al treatments and among pH values were determined using two‐way analysis of variance (ANOVA) and simple linear regression, respectively. Significance of correlations between Al and other minerals was determined using Pearson correlation analysis, while correcting for multiple comparisons. The *q*‐values were calculated for multiple testing using the Benjamini–Hochberg false discovery rate (Benjamini & Yosef, 1995) from the *p*‐values obtained in the correlation analysis, performed using the “corr.test” function of the R package “psych” ver. 1.9.12.31 (Revelle, 2020). The *q‐*values < .05 were considered significant. The data in the figures are the mean ± SD of three biological replicates.

The individual values of each treatment were used for multivariate analysis of the ionome, quantifying the data for 14 elements without Al. Data were normalized by calculating *z*‐score values for each mineral in principal component analysis (PCA). The PCA was performed using the R function “prcomp,” and the principal component scores and biplots were plotted using the R package “ggplot2” ver. 3.1.0 (Wickham, 2016), “cowplot” ver. 1.0.0 (Wilke, 2019), “factoextra” ver. 1.0.7 (Kassambara & Mundt, 2020), and “FactoMineR” ver. 2.3 (Husson, Josse, Le, & Mazet, 2020). Significant differences in ionome profiles between pH and Al treatments were determined using permutational multivariate analysis of variance (PERMANOVA). PERMANOVA was performed using the “adonis” function of the R package “vegan” ver. 2.5‐6 (Oksanen et al., 2019).

## 3 RESULTS

### 3.1 Growth in different pH and Al treatments

**FIGURE 1:**
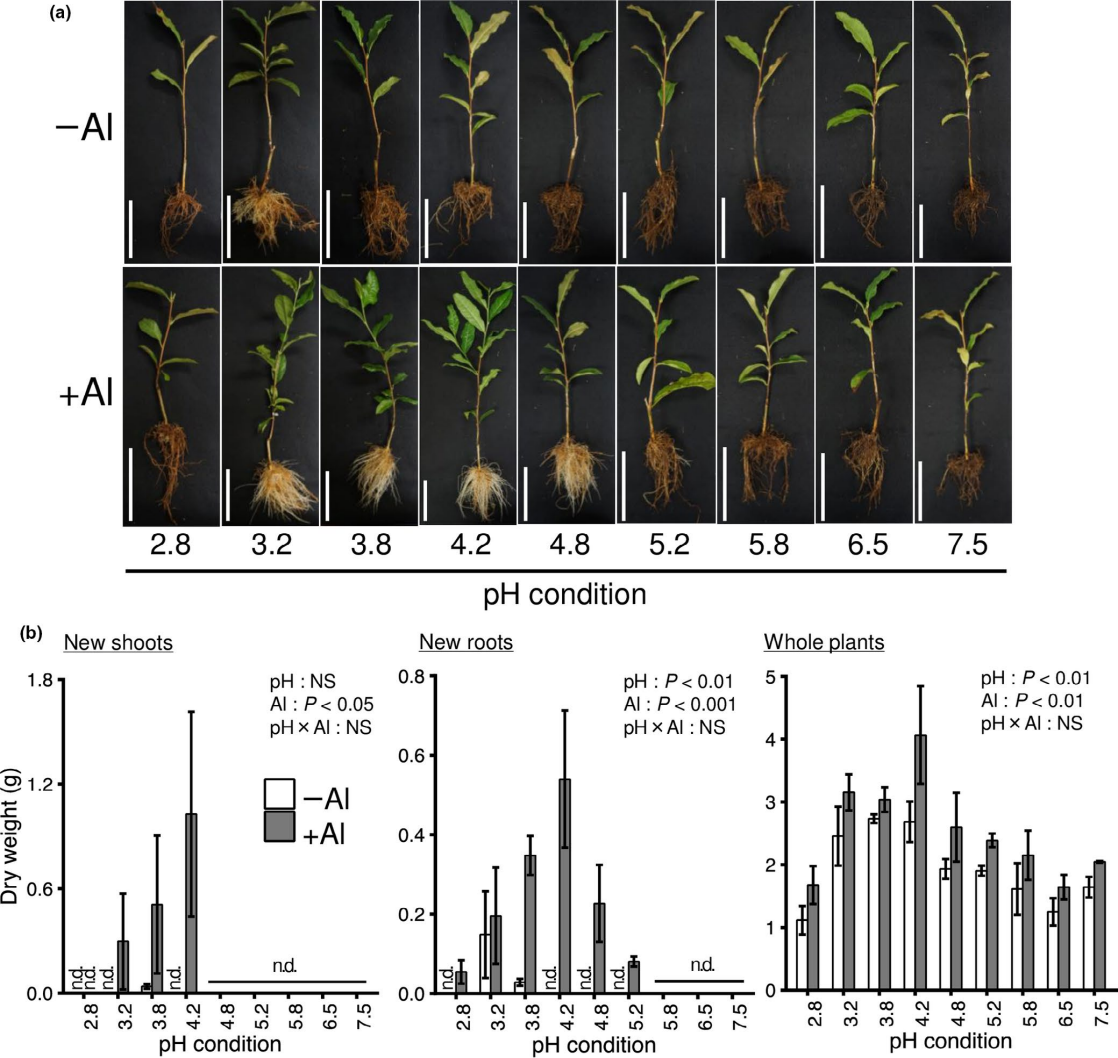
Effects of pH and Al treatment on the growth of 1‐year‐old rooted cuttings of tea plants. Phenotypes after growth for 5 weeks under the treatments (a). Dry weight of new shoots (left), new roots (middle), and whole plants (right) (b). Data and error bars are the mean ± SD (*n* = 3). Statistical tests for significant differences by two‐way ANOVA are shown in the figures. NS: not significant (*p* > 0.05); n.d.: no data. Bar = 10 cm (a)

We evaluated the effects of pH and Al on growth using 1‐year‐old rooted tea cuttings (Figure S1). The development of new shoots was observed only at pH 3.2, 3.8, and 4.2 with Al treatment (+Al), and there was no shoot growth without Al treatment (−Al) (Figure 1a,b). The development of new roots was also observed at pH 2.8–5.2 in +Al and pH 3.2–3.8 in –Al treatments. The growth of new roots and whole plants was significantly affected by pH and Al (Figure 1a,b; two‐way ANOVA, *p* < .001 or < .01). Root growth stimulation by Al was observed at pH 3.2–5.2 (Figure 1b). Superior growth of shoots and roots was observed at pH 4.2 in the +Al treatment (Figure 1a,b).

**FIGURE 2:**
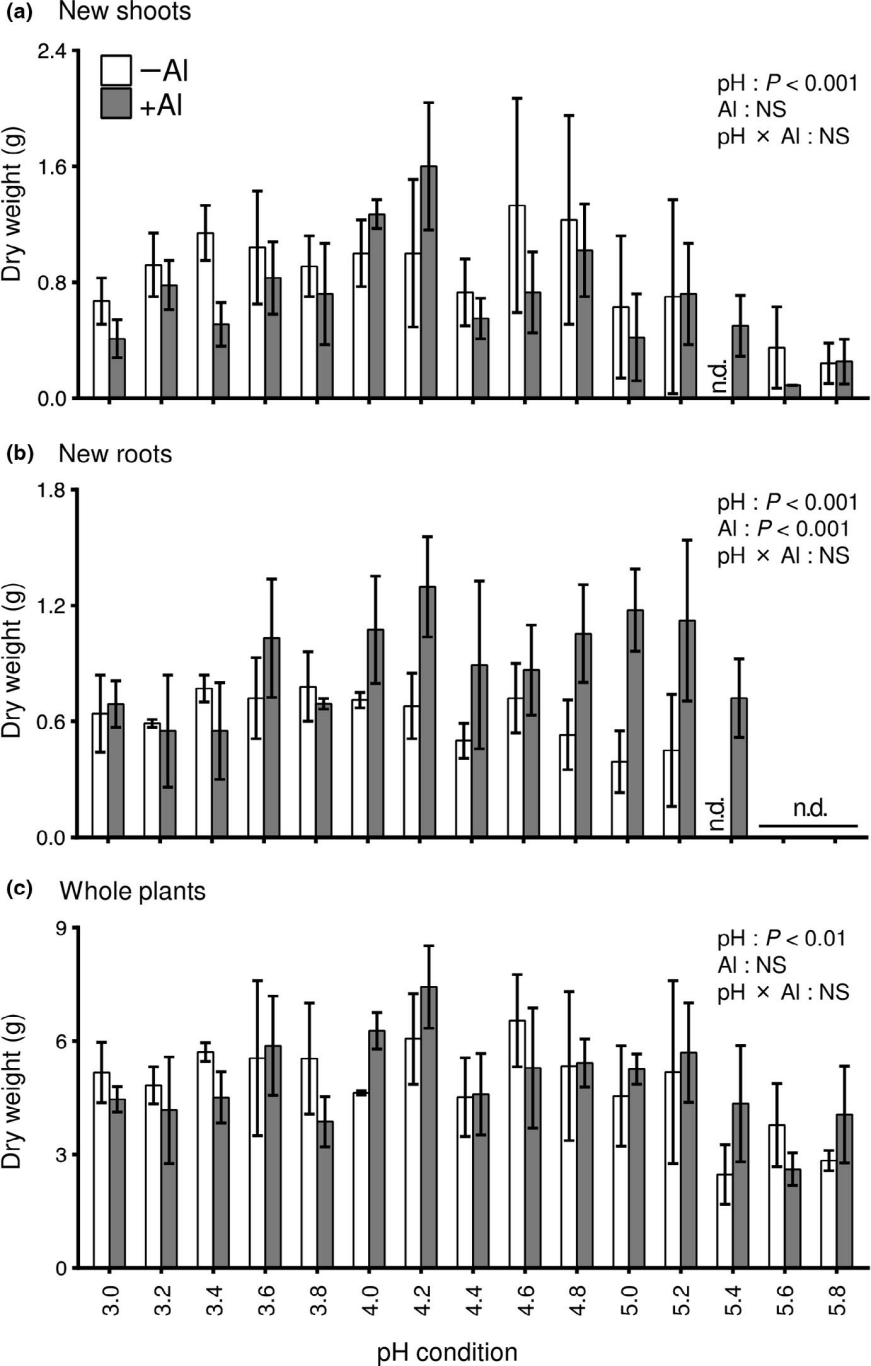
Effects of pH and Al treatment on the growth of prematured rooted cuttings of tea plants. Dry weight of new shoots (a), new roots (b), and whole plants (c) after growth for 2 months under the treatments. Data and error bars are the mean ± SD (*n* = 3). Statistical tests for significant differences by two‐way ANOVA are shown in the figures. NS: not significant (*p* > 0.05)

In the second experiment, we evaluated the effects of pH and Al on the growth of prematured 1‐year‐old rooted tea cuttings. The development of new shoots was observed at pH 3.0–5.2 regardless of Al treatment, and there was no significant difference between the Al treatments (Figure 2a; two‐way ANOVA, *p *> .05). The development of new roots in the –Al and +Al treatments was observed at pH 3.0–5.2 and 3.0–5.4, respectively (Figure S2). The growth of new roots was significantly affected by pH and Al (Figure 2b; two‐way ANOVA, *p* < .001). Root growth stimulation by Al was observed especially at pH 4.2 and 5.0 (Figure 2b). Superior growth of shoots and roots was observed at pH 4.2 in the +Al treatment (Figure 2). In both hydroponic experiments, the growth of tea plants was highest at pH 4.2 in the +Al treatment.

### 3.2 Al content and accumulation in response to pH treatment

**FIGURE 3:**
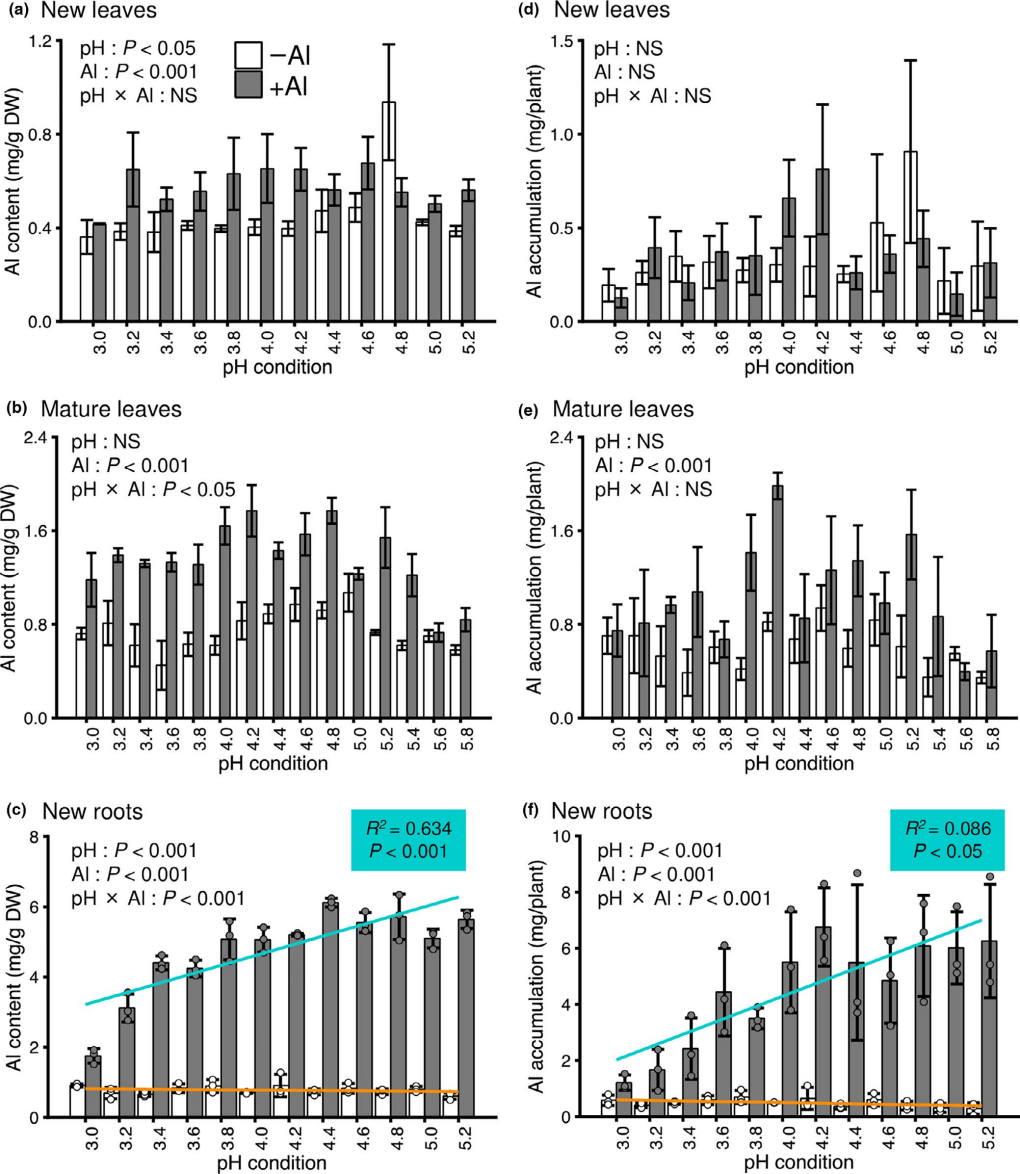
Effect of pH and Al treatment on the Al content and accumulation in the predominant mineral‐accumulating tissues of tea plants. Al content and accumulation in young leaves (a, d), mature leaves (b, e), and young roots (c, f) as the major accumulation organs. Data and error bars are the mean ± SD (*n* = 3). Statistical tests for significant differences by two‐way ANOVA are shown in the figures. In (c) and (f), results of simple linear regression analysis are shown in the cyan box. NS; not significant (*p* > 0.05)

We analyzed the Al content and accumulation in new leaves, mature leaves, and new roots, as the predominant tissues for mineral accumulation in tea plants, under different pH conditions. The Al content in the three tissues was increased by Al treatment (Figure 3a–c; two‐way ANOVA, *p* < .001), and especially under the optimum hydroponic growth conditions at around pH 4.2. The Al accumulation in mature leaves and new roots was increased by Al treatment (Figure 3d–f; two‐way ANOVA, *p* < .001). The Al content and accumulation were higher in new roots, mature leaves, and new leaves (in descending order; Figure 3). In new roots, the Al content and accumulation were affected by pH (Figure 3c,f; two‐way ANOVA, *p* < .001) and lowered with decrease in pH (Figure 3c,f; simple linear regression test, *p *< .001 and < .05, respectively). The highest Al content at pH 4.4 in new roots was approximately threefold that observed at pH 3.0.

### 3.3 Tissue ionome dynamics in response to pH and Al treatment

**FIGURE 4:**
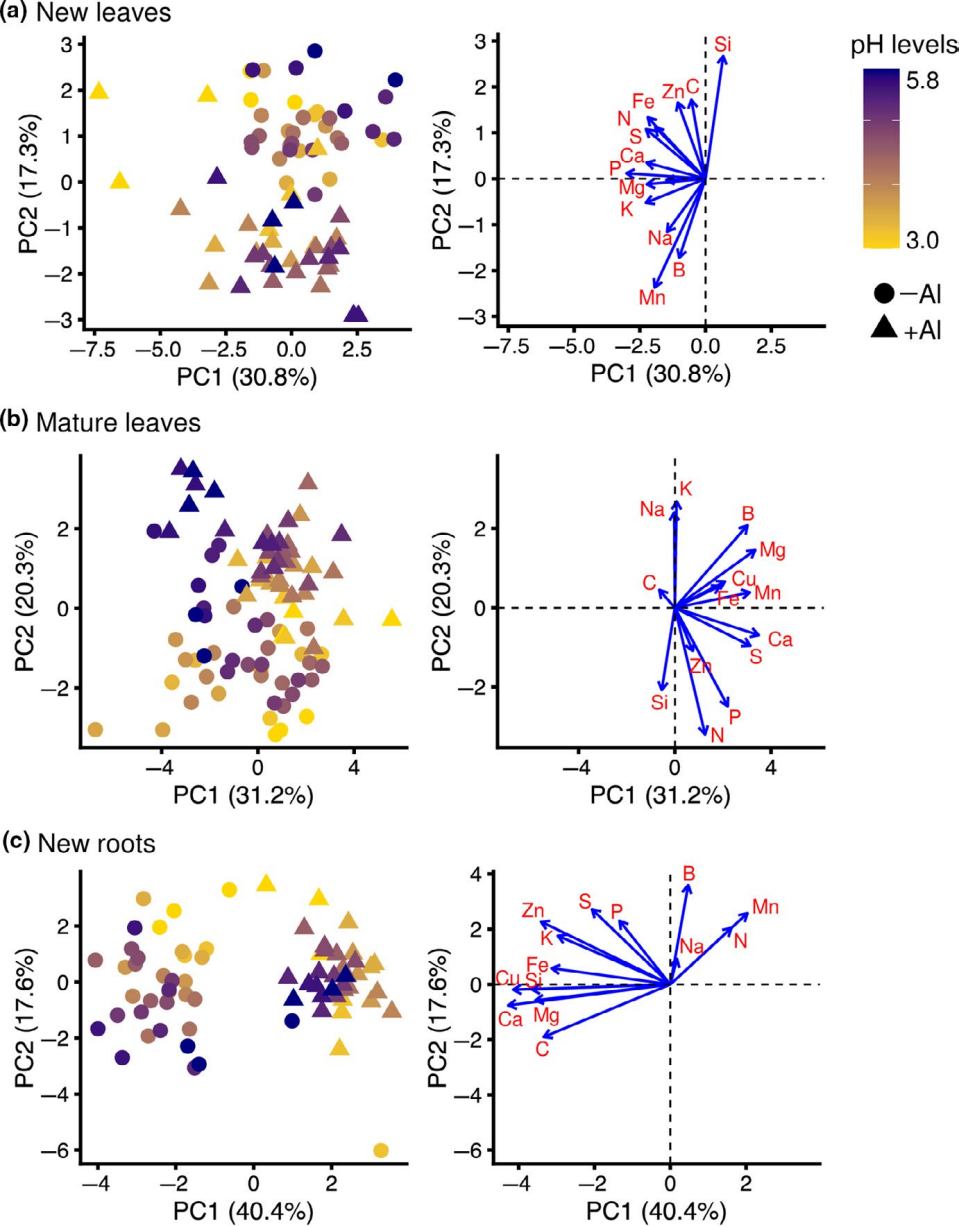
Principal component analysis (PCA) of ionome data under different pH values with and without Al treatment. Scores for PC1 and PC2 in ionome profiles of new leaves (a), mature leaves (b), and new roots (c), and the corresponding loading factors, respectively. The pH of each sample is represented on a color gradient, yellow to magenta, denoting low to high pH, respectively. Treatments with or without Al are shown as triangles and circles, respectively. Data for the following 14 elements without Al were normalized and used as ionome data for PCA: Fe, Na, B, P, S, Si, Ca, Cu, K, Mg, Mn, Zn, N, and C

**FIGURE 5:**
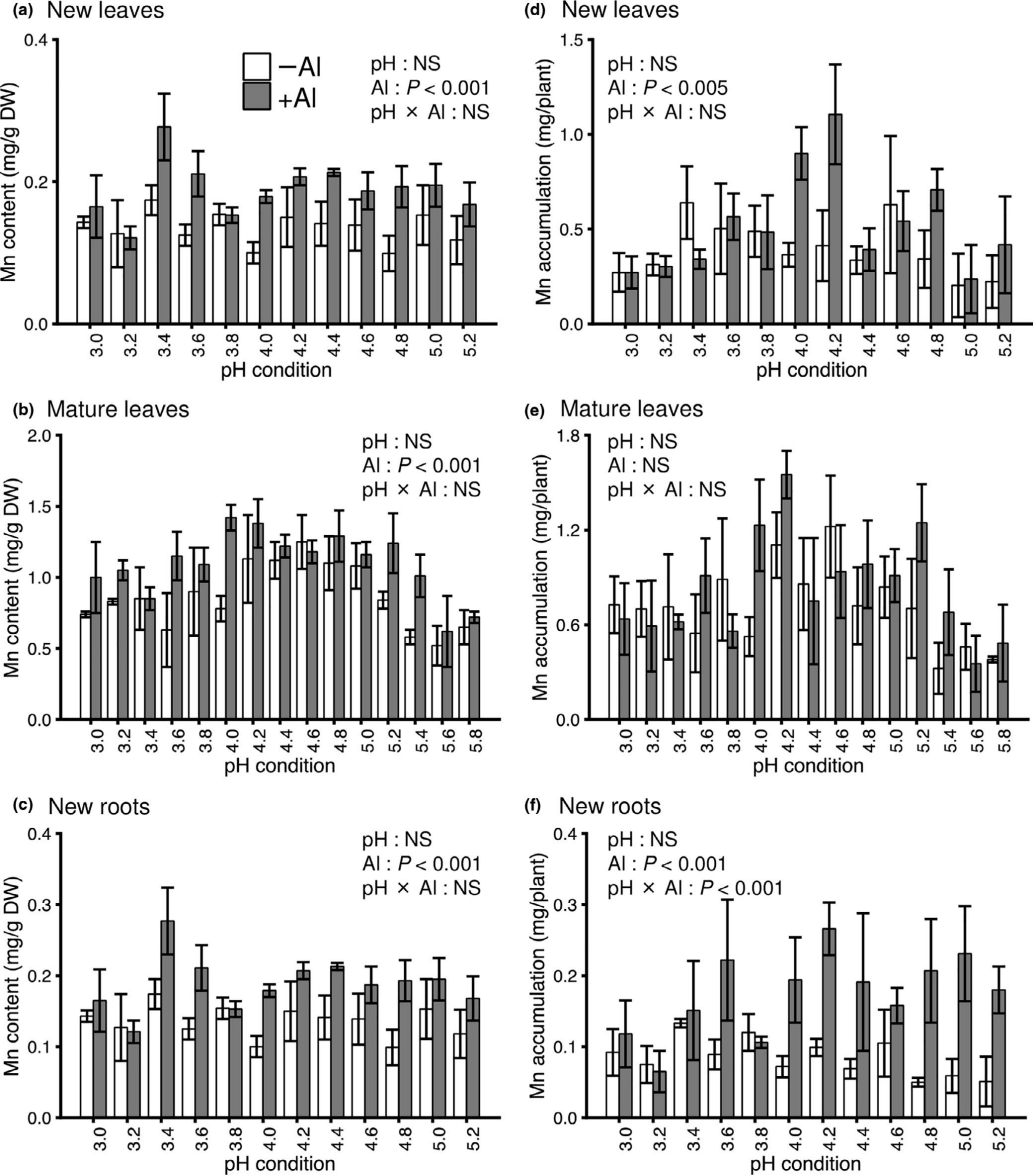
Effects of pH and Al treatment on the Mn content and accumulation in the predominant mineral‐accumulating tissues of tea plants. Al content and accumulation in young leaves (a, d), mature leaves (b, e), and young roots (c, f) as the major accumulation organs. Data and error bars are the mean ± SD (*n* = 3). Statistical tests for significant differences by two‐way ANOVA are shown in the figures. NS; not significant (*p* > 0.05)

To determine the effect of pH and Al on the distribution of a range of elements in the predominant mineral‐accumulating tissues, we used ICP‐OES and a CN analyzer to analyze the following 14 elements, in addition to Al, constituting the ionome: Fe, Na, B, P, S, Si, Ca, Cu, K, Mg, Mn, Zn, N, and C (Table S1). PERMANOVA revealed that the ionome profiles in all the three tissues tested were significantly affected by pH and Al treatment (Table 1). In the PERMANOVA analysis, pH treatment explained 7.2%, 10.6%, and 8.0%, whereas Al treatment explained 14.8%, 16.7%, and 54.3% of the total variability in new leaves, mature leaves, and new roots, respectively (Table 1). PCA showed a clear separation of ionome profiles not by pH treatment but by the presence/absence of Al in all the three tissues tested, and loading factor biplots identified the elements that contributed to that separation (Figure 4). In new leaves, the difference in ionome profiles with Al treatment was observed as the second principal component (PC2; Figure 4a), representing 19.6% of the total variation. The predominant elements that contributed to PC2 were Mn, B, Si, and Fe (Figure 4a). In mature leaves, the difference in ionome profiles with Al treatment was also observed as PC2 (Figure 4b), representing 24.7% of the total variation. The predominant elements that contributed to PC2 were N, B, Mg, and Mn (Figure 4b). In new roots, the difference in ionome profiles with Al treatment was observed as the first principal component (PC1; Figure 4c), representing 46.2% of the total variation. The predominant elements that contributed to PC1 were Ca, Cu, C, Mg, and Mn (Figure 4c). These results were statistically supported by two‐way ANOVA (Table 2). The contents of cationic nutrients in new roots, such as Ca, Cu, Mg, Fe, K, and Zn, were decreased by Al treatment (Figure 4c). In contrast, only Mn, despite being a cationic nutrient, was significantly increased in content in the three tissues tested only by Al treatment (Figure 5a–c; two‐way ANOVA, *p* < .001), especially around the optimum pH 4.2 for the growth of tea plants. An Al‐induced increase in Mn accumulation in new leaves and new roots was also observed (Figure 5d–f, two‐way ANOVA, *p* < .005 and < .001, respectively).

**TABLE 1:** Statistical tests by permutational analysis of variance (PERMANOVA) for the ionome profile under acidic pH levels with and without Al

Tissues	Factors	*p*‐value	*R* ^2^ value
New leaves	pH	<.001	.072
	Al	<.001	.148
	pH × Al	<.005	.037
	Residuals	−	.743
Mature leaves	pH	<.001	.106
	Al	<.001	.167
	pH × Al	<.001	.037
	Residuals	−	.690
New roots	pH	<.001	.080
	Al	<.001	.334
	pH × Al	<.001	.044
	Residuals	−	.543

**TABLE 2:** Statistical test results by two‐way ANOVA results for the ionome data under acidic pH levels with and without Al

Tissues	Factors	Al	C	N	P	K	Ca	Mg	S	B	Fe	Mn	Zn	Cu	Na	Si
New leaves	pH	<.05	NS	NS	<.001	NS	< .001	NS	<.001	<.05	NS	NS	NS	NS	NS	<.01
	Al	<.001	NS	NS	<.01	<.05	NS	NS	NS	<.001	NS	<.001	NS	NS	<.001	<.001
	pH × Al	NS	<.001	NS	NS	NS	NS	<.01	NS	NS	NS	NS	NS	NS	NS	NS
Mature leaves	pH	NS	<.05	<.001	<.001	<.001	<.01	NS	<.001	NS	NS	NS	<.001	NS	<.05	<.001
	Al	<.001	NS	<.001	<.001	<.001	NS	<.001	NS	<.001	NS	<.001	NS	NS	<.01	<.001
	pH × Al	<.05	<.01	NS	<.01	<.05	NS	<.05	NS	<.05	<.01	NS	<.05	NS	NS	<.001
New roots	pH	<.001	<.001	NS	<.001	NS	<.001	<.001	NS	NS	<.001	NS	NS	<.05	NS	<.01
	Al	<.001	<.001	<.05	<.01	<.001	<.001	<.001	<.05	NS	<.001	<.001	<001	<.001	NS	<.001
	pH × Al	<.001	NS	NS	<.001	NS	<.01	<.05	<.001	NS	NS	NS	<.01	<.05	NS	<.001

Note

Values in table mean a threshold of *p*‐value by two‐way ANOVA.

Abbreviation: NS, not significant (*p* > 0.05).

### 3.4 **Correlation** **between Al and other minerals**

**FIGURE 6:**
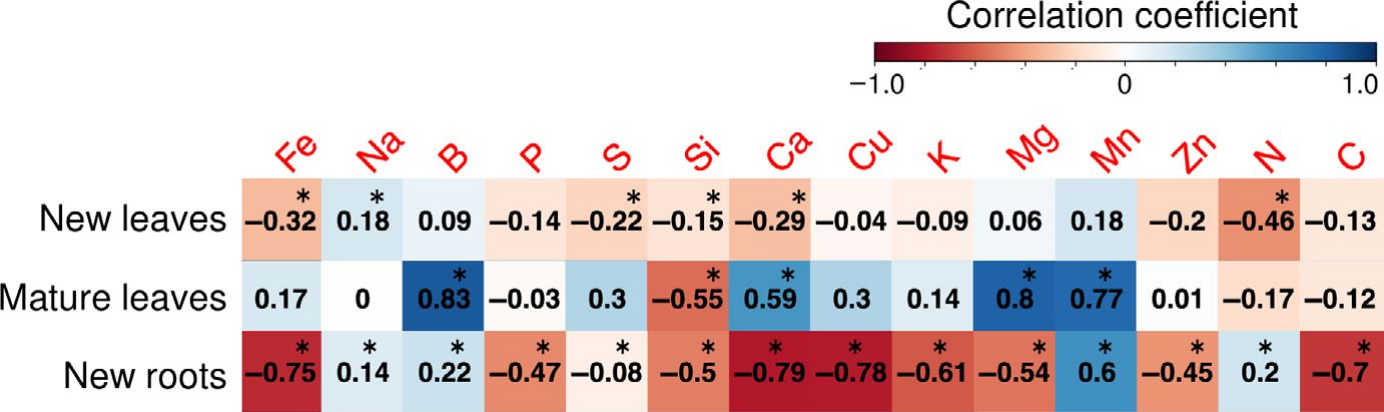
Correlation coefficient between Al and other minerals in the predominant mineral‐accumulating tissues of tea plants. Asterisks indicate significant correlations with Al. Significance of correlation between Al and other minerals was determined by statistical test for correlation correcting for multiple testing using the Benjamini–Hochberg false discovery rate (BH‐FDR, *q* < 0.05)

Correlation analyses of the ionome dataset revealed positive and negative correlations among the 15 elements in each of the three mineral‐accumulating tissues (Figure S3). To understand the relationship between the contents of Al and each mineral, we focused the analysis to correlations between Al and the other minerals (Figure 6). The minerals correlated with Al were differed in each of the three tissues tested but some similarities were observed, as follows. Na was positively correlated with Al in both new leaves and new roots (Figure 6). B and Mn were positively correlated with Al in mature leaves and new roots (Figure 6). Si was negatively correlated with Al in the three tissues. Ca and Fe were negatively correlated with Al in new leaves and new roots (Figure 6).

## 4 DISCUSSION

The major rhizotoxicity factor in acid soils is the excess of H^+^ and Al^3+^, which causes inhibition of root growth and nutrient uptake (Kochian et al., 2004). However, tea plants can grow vigorously in acidic soil. Furthermore, in the presence of Al, the growth of tea plants is not inhibited but rather stimulated (Ghanati et al., 2005; Konishi et al., 1985; Morita et al., 2008; Sun et al., 2020). This phenomenon was observed in the present study (Figures 1 and 2), with our results confirming that Al was beneficial to the growth of tea plants. Hydroponic experiments under diverse acid pH conditions revealed that tea plants grew well at pH 4.0–5.4, and especially around pH 4.2. Although tea plants were able to survive under severe acidic conditions, such as pH less than 4.0, growth was inferior to that at the optimum pH 4.2. However, at pH 2.8–3.0, tea plants did not grow even in the presence of Al, and new leaves and roots did not develop or grow (Figure 1). In some Japanese tea fields, heavy application of N fertilizer has caused severe soil acidification to around pH 3.0 (Tokuda & Hayatsu, 2004). Tachibana et al. (1995) reported that the soil pH of 126 tea fields in Mie Prefecture, a major Japanese green tea cultivation region in Japan, ranged from pH 2.9 to 5.9, with that of most fields less than pH 4.0. The present results indicate that the soil pH is important for improvement of tea production and quality. The optimum pH for the growth of tea plants in hydroponic culture was around pH 4.2, with inferior growth under pH less than 3.8 or higher than 5.0. In addition, under the optimum pH conditions, the growth stimulation by Al was pronounced (Figures 1 and 2).

The Al content and accumulation were increased by Al treatment, and this was observed especially under the optimal conditions for the growth of tea plants around pH 4.2 (Figure 3). These results suggest that tea plants actively absorbed and accumulated Al under the optimum acidic pH for growth. In new roots, the Al content and accumulation were affected by pH and declined with decrease in pH (Figure 3c). These results suggest that excessive H^+^ competitively inhibited Al uptake in the roots of tea plants.

Ionomics is a useful tool to understand changes in physiological processes in response to nutrient status (Baxter et al., 2008). The alteration to ionome dynamics was confirmed in response to environmental factors (Chu et al., 2016; Quadir et al., 2011; Wu et al., 2013). In acidic soil, Al affects the cellular homeostasis of various ions, resulting in the inhibition of plant growth (Babourina & Rengel, 2009; Bose et al., 2010a, 2010b, 2011, 2013; Plieth et al., 1999; Rengel & Zhang, 2003). However, few studies have investigated the effect of Al as a beneficial element on the ionome in species that are well adapted to acid soils, such as tea plants. To reveal the effect of pH and Al on the plant ionome, we measured and analyzed 14 elements in addition to Al in new leaves, mature leaves, and new roots as the predominant mineral‐accumulating tissues of tea plants. PERMANOVA and PCA revealed the alteration to ionome profiles in the three predominant mineral‐accumulating tissues not in response to pH but induced by Al, and loading factor biplots identified the elements that contributed to that alteration (Table 1, Figure 4). Ionome profiles in new roots were drastically altered by Al treatment but not by pH, and to a greater extent than in new and mature leaves (Figure 4). In new roots, the contents of most cationic nutrients, such as Ca, Cu, Mg, Fe, K, and Zn, were decreased by Al treatment (Figure 4C, Table 2 and Table S1). It has been considered that the activity of these cationic nutrients at the cell membrane surface might compete with excess Al^3+^ (Kinraide et al., 1992). However, in leaves the contents of these elements were not affected (Figure 4a,b, Table 2 and Table S1). These results suggest that Al suppressed the absorption of many cationic elements, but in its beneficial role it might aid their efficient translocation from the root to the shoot in tea plants. Therefore, Al might complement the nutrient functions of these cationic elements and enable good growth in poor nutrient environments such as acid soils. However, to clarify the possible nutritional roles of Al in tea plants, further physiological experiments are needed.

Interestingly, the only element significantly increased in content by Al treatment was Mn, despite it being a cationic nutrient; Mn content was not affected by pH conditions, and an Al‐induced increase in Mn accumulation in the leaves was also observed (Figure 5). In rice, which is the most tolerant to Al among the small‐grained cereal crops (Foy, 1998), Al alleviated Mn toxicity, which was attributed to decreased shoot Mn accumulation resulting from an Al‐induced decrease in root symplastic Mn uptake (Wang et al., 2015). This phenomenon of Al‐induced decrease in Mn uptake has been observed in other plant species (Blair & Taylor, 1997; Clark, 1977; Taylor, Blamey, & Edwards, 1998; Yang, You, & Xu, 2009). The decrease in root symplastic Mn uptake results from an Al‐induced change in cell membrane potential according to the Gouy–Chapman–Stern model (Kinraide, Yermiyahu, & Rytwo, 1998; Kopittke, Wang, Menzies, Naidu, & Kinraide, 2014; Wang et al., 2015). Therefore, in tea plants, in contrast to other plant species, Al might enhance Mn uptake and translocation via a Mn transporter, unlike the decrease in Mn electrical activity in the cell membrane induced by Al. Given that toxicity caused by excess Mn occurs in acidic soils in the same manner as Al toxicity (Kochian et al., 2004), both Al and Mn stress‐adaptation mechanisms might develop simultaneously in acidic soils through coordinated adaptation processes. To understand the mechanism of adaptation to acidic soils, further verification of the interaction between these two mineral stresses is a topic for future research.

Correlation analysis confirmed the results revealed by PCA (Figure 6). B and Mn were positively correlated with Al in both mature leaves and new roots, which are the predominant Al‐accumulating tissues in tea plants (Figure 6). Hajiboland et al. (2015) reported Al‐induced increases in the contents of B in the root cell wall (CW) and of CW‐bound phenolic acids, but not of lignin, and suggested that increased B partitioning to the CW and reduced lignification were important components in the growth stimulation by Al. The present analysis also confirmed interactions between Al and B.

**FIGURE 7:**
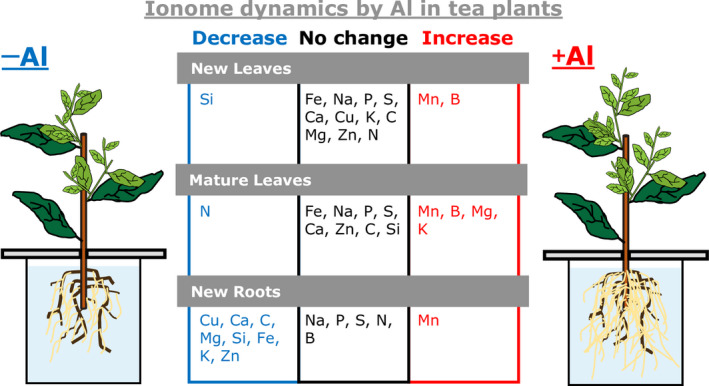
Overview of the Al‐responsive tissue ionome in tea plants

In conclusion, we revealed that the growth of tea plants was stimulated by both Al and acidic pH, with optimum growth observed in a narrow range around pH 4.2 and inferior growth at pH less than 3.8 or higher than 5.0. Under the optimum pH conditions, Al markedly stimulated growth and Al accumulation at the whole‐plant scale. Furthermore, we showed that the alteration to ionome profiles caused by Al in tea plants did not depend on pH (Figure 7). Our findings indicated that the distinct alterations in tissue ionome in tea plants were possibly attributable to the development of adaptations to acid soils. Through integration of the present results with other omics data, such as genome, transcriptome, and metabolome data, and use of phenotypes associated with genetic variation, these findings will accelerate progress in understanding the roles of Al as a beneficial element for some species, such as tea plants, that are well adapted to acid soils. Recently, the draft genomes of the two important tea varieties, *C. sinensis* var. *sinensis* (Wei et al., 2018) and var. *assamica* (Xia et al., 2017), were sequenced using a next‐generation sequencing platform. Next‐generation sequencing technologies are accelerating the application of transcriptome analysis in tea plants; for example, use of RNA‐sequencing enables advances in elucidation of various environmental responses in different genotypes (Bai et al., 2019; Li et al., 2015; Li, Xiang, et al., 2017; Li, Huang, et al., 2017; Lu et al., 2018). Recently, Li, Huang, et al. (2017) reported an Al‐responsive de novo RNA‐sequencing transcriptome analysis of tea roots that indicated common and distinct Al‐tolerance mechanisms between tea plants and rice, Arabidopsis, and buckwheat. The present findings provide a foundation for the nutritional knowledge needed to clarify the role of Al as a beneficial.

## Author contributions

H.Y. conducted the hydroponic growth tests and harvested the samples. H.Y., Y.F., and S.Y. performed the mineral analysis. H.Y. and T.I. performed the ionome data analysis. A.M. and T.I. acquired the funding. H.Y., A.M., and T.I. designed the experiments and wrote the manuscript. All the authors read and approved the manuscript.

## Conflict of interest

The authors declare no conflict of interest associated with this manuscript.

## Supporting information

Figure S1–S3

Table S1

## Data Availability

The data that supports the findings of this study are included in the article and supplementary material.
