# Supplementary material for: Tissue ionome response to rhizosphere pH and aluminum in tea plants (Camellia sinensis L.), a species adapted to acidic soils
Source: Plant Environ Interact. 2020 Aug 10;1(2):152–64. doi: 10.1002/pei3.10028 (PMC10168086; doi:10.1002/pei3.10028)
Supplement: Supplementary file 1 — Figure S1–S3 [file PEI3-1-152-s002.pptx]

## Slide 1
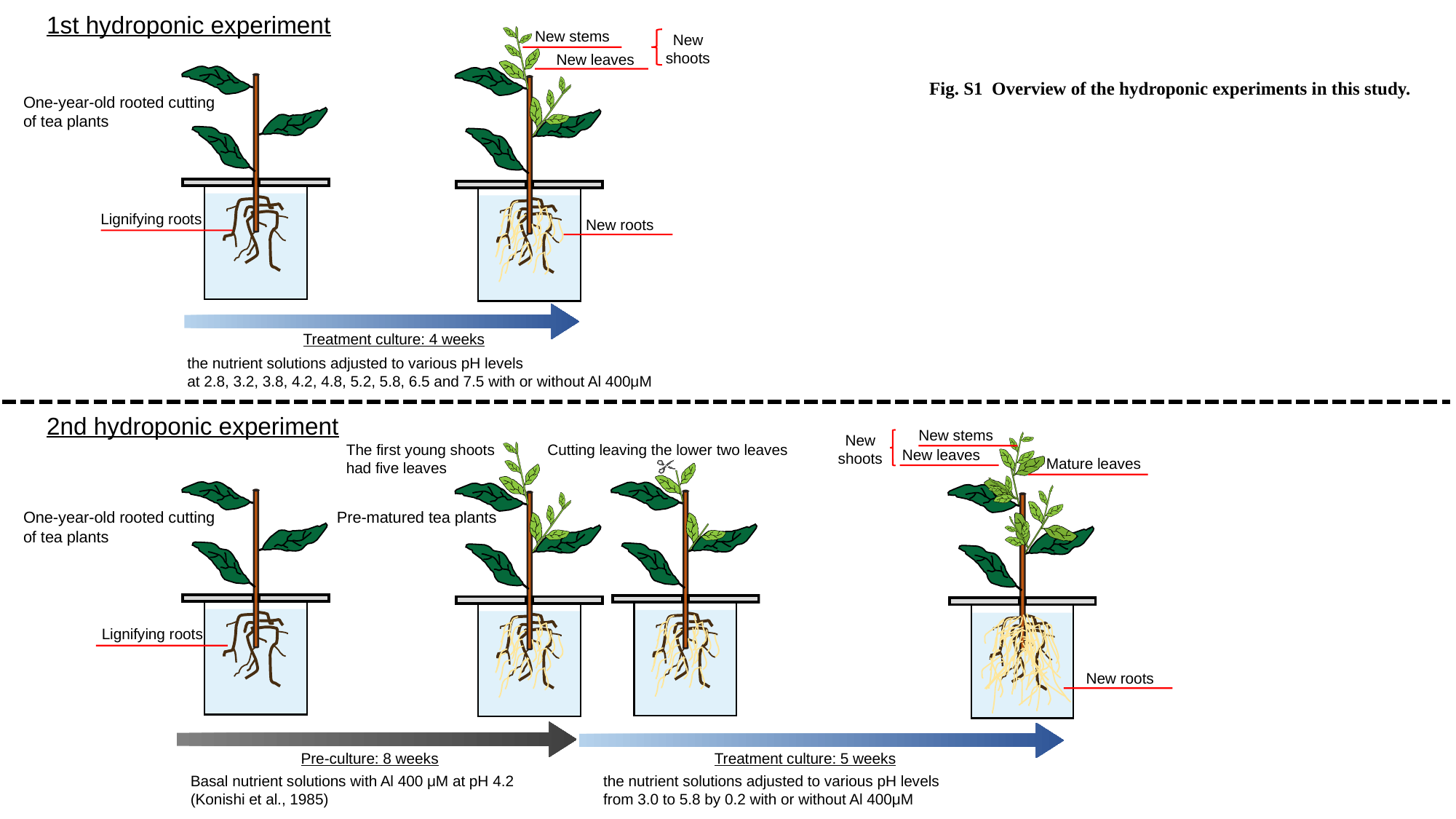

1st hydroponic experiment
New stems
New
shoots
New leaves
Fig. S1 Overview of the hydroponic experiments in this study.
One-year-old rooted cutting
of tea plants
Lignifying roots
New roots
Treatment culture: 4 weeks
the nutrient solutions adjusted to various pH levels
at 2.8, 3.2, 3.8, 4.2, 4.8, 5.2, 5.8, 6.5 and 7.5 with or without Al 400μM
2nd hydroponic experiment
New stems
New
shoots
Cutting leaving the lower two leaves
The first young shoots had five leaves
New leaves
Mature leaves
Pre-matured tea plants
One-year-old rooted cutting
of tea plants
Lignifying roots
New roots
Pre-culture: 8 weeks
Treatment culture: 5 weeks
Basal nutrient solutions with Al 400 μM at pH 4.2
(Konishi et al., 1985)
the nutrient solutions adjusted to various pH levels from 3.0 to 5.8 by 0.2 with or without Al 400μM

## Slide 2
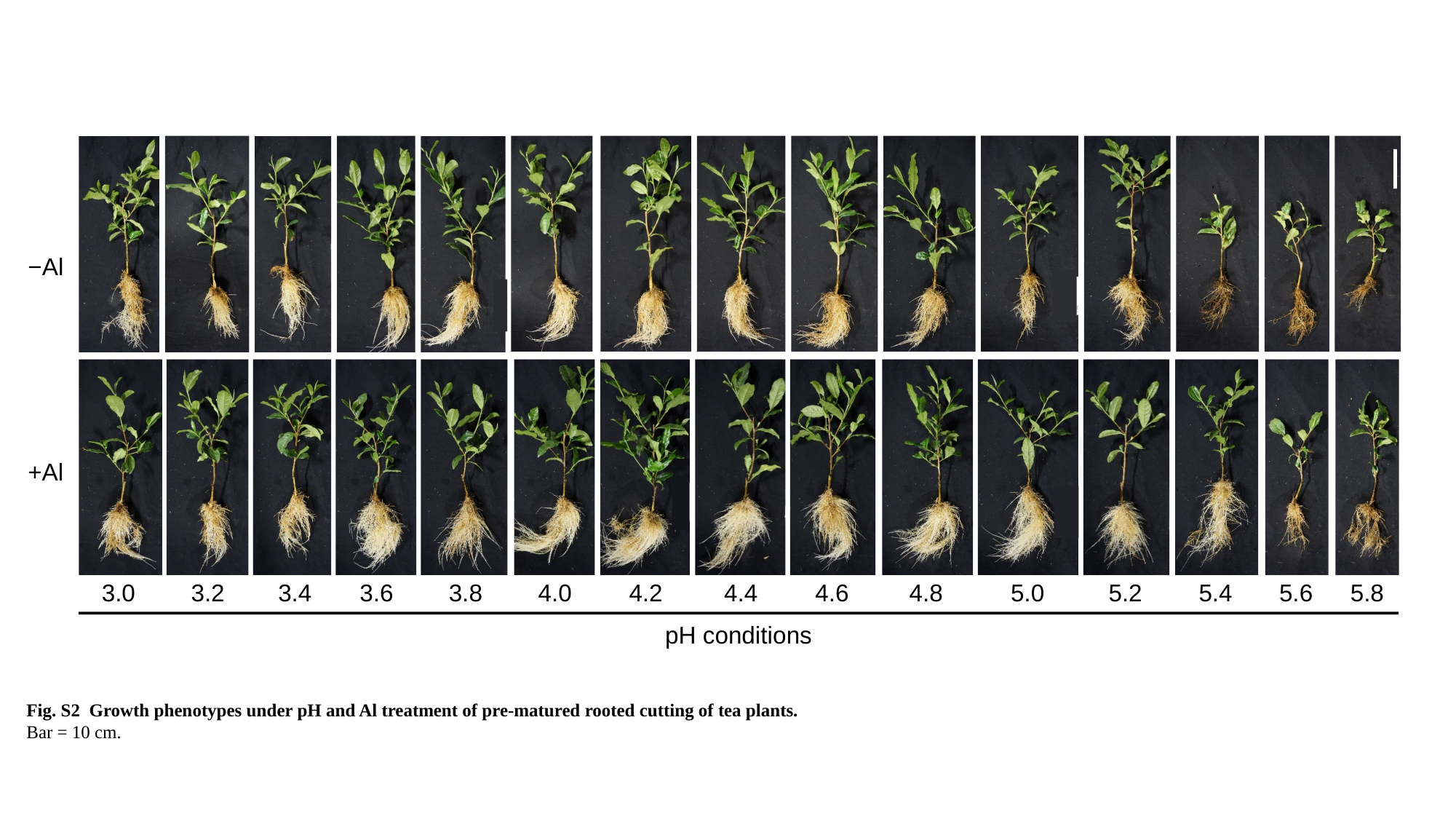

−Al
+Al
3.0
3.2
3.4
3.6
3.8
4.0
4.2
4.4
4.6
4.8
5.0
5.2
5.4
5.6
5.8
pH conditions
Fig. S2 Growth phenotypes under pH and Al treatment of pre-matured rooted cutting of tea plants.
Bar = 10 cm.

## Slide 3
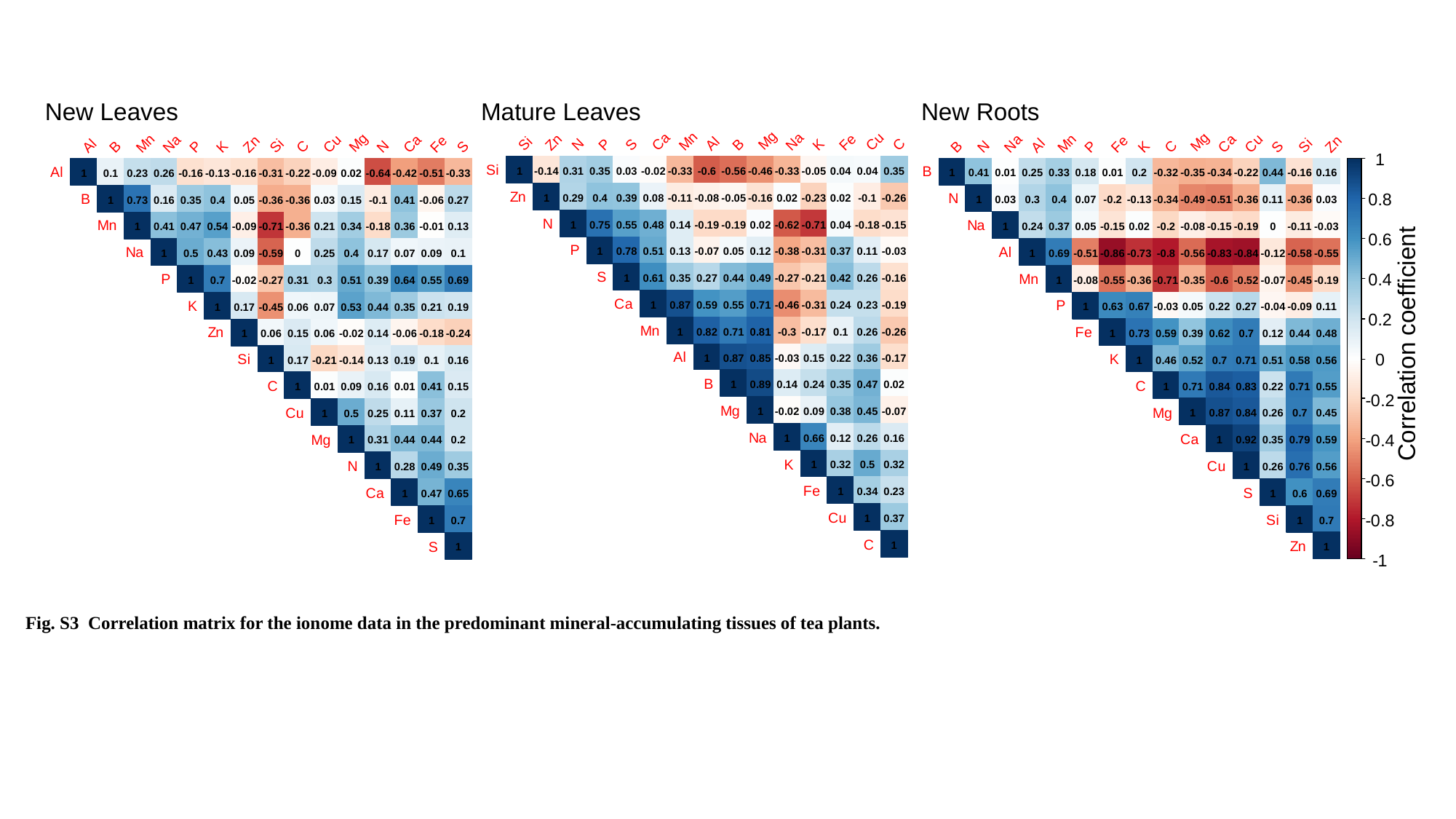

New Leaves
Mature Leaves
New Roots
Correlation coefficient
Fig. S3 Correlation matrix for the ionome data in the predominant mineral-accumulating tissues of tea plants.
